# Author Correction: A microRNA program regulates the balance between cardiomyocyte hyperplasia and hypertrophy and stimulates cardiac regeneration

**DOI:** 10.1038/s41467-022-32785-0

**Published:** 2022-08-25

**Authors:** Andrea Raso, Ellen Dirkx, Vasco Sampaio-Pinto, Hamid el Azzouzi, Ryan J. Cubero, Daniel W. Sorensen, Lara Ottaviani, Servé Olieslagers, Manon M. Huibers, Roel de Weger, Sailay Siddiqi, Silvia Moimas, Consuelo Torrini, Lorena Zentillin, Luca Braga, Diana S. Nascimento, Paula A. da Costa Martins, Jop H. van Berlo, Serena Zacchigna, Mauro Giacca, Leon J. De Windt

**Affiliations:** 1 Department of Molecular Genetics, Faculty of Science and Engineering, Faculty of Health, Medicine and Life Sciences, Maastricht University, Maastricht, The Netherlands; 2 i3S - Instituto de Investigação e Inovação em Saúde, INEB - Instituto Nacional de Engenharia Biomédica, ICBAS - Instituto de Ciências Biomédicas de Abel Salazar, University of Porto, Porto, Portugal; 3 Department of Molecular Genetics, Erasmus University MC, Rotterdam, The Netherlands; 4 The Abdus Salam International Centre for Theoretical Physics, Trieste, Italy; 5 IST Austria, Klosterneuburg, Austria; 6 Stem Cell Institute and Lillehei Heart Institute, Department of Medicine, University of Minnesota, Minneapolis, MN USA; 7 Department of Pathology, University Medical Center Utrecht, Utrecht, The Netherlands; 8 Department of Cardiothoracic Surgery, Radboud University Medical Center, Nijmegen, The Netherlands; 9 International Centre for Genetic Engineering and Biotechnology (ICGEB), Trieste, Italy; 10 Department of Physiology and Cardiothoracic Surgery, Faculty of Medicine, University of Porto, Porto, Portugal; 11 School of Cardiovascular Medicine and Sciences, King’s College London, London, UK

**Keywords:** miRNAs, Cardiac regeneration

Correction to: *Nature Communications* 10.1038/s41467-021-25211-4, published online 10 August 2021

In the original version of this Article, the magnified image of the hematoxylin and eosin (H&E) staining in the Figure 1d column labelled “TAC knockout’ was inadvertently replaced with a duplicate of the magnified image of the same staining from the Figure 1d column labelled “TAC wild-type”. This has been corrected in both the PDF and HTML versions of the Article.

