# Supplementary material for: A microRNA program regulates the balance between cardiomyocyte hyperplasia and hypertrophy and stimulates cardiac regeneration
Source: Nat Commun. 2021 Aug 10;12:4808. doi: 10.1038/s41467-021-25211-4 (PMC8355162; doi:10.1038/s41467-021-25211-4)
Supplement: Supplementary file 1 — Supplementary Information File [file 41467_2021_25211_MOESM1_ESM.pdf]

A microRNA program regulates the balance between cardiomyocyte hyperplasia and hypertrophy and stimulates cardiac regeneration

[1] Andrea Raso; [1] Ellen Dirkx; [1,2] Vasco Sampaio-Pinto; [1,3] Hamid el Azzouzi; [4,5] Ryan J. Cubero; [6] Daniel W. Sorensen; [1] Lara Ottaviani; [1] Servé Olieslagers; [7] Manon M. Huibers; [7] Roel de Weger; [8] Sailay Siddiqi; [9] Silvia Moimas; [9] Consuelo Torrini; [9] Lorena Zentillin; [9] Luca Braga; [2] Diana S. Nascimento; [1,10] Paula A. da Costa Martins; [6] Jop H. van Berlo; [8] Serena Zacchigna; [9,11] Mauro Giacca and [1] Leon J. De Windt*

[1]Department of Molecular Genetics, Faculty of Science and Engineering, Faculty of Health, Medicine and Life Sciences, Maastricht University, 6229 ER Maastricht, The Netherlands;
[2]i3S - Instituto de Investigação e Inovação em Saúde, INEB - Instituto Nacional de Engenharia Biomédica, ICBAS - Instituto de Ciências Biomédicas de Abel Salazar, University of Porto, Porto, Portugal.
[3]Department of Molecular Genetics, Erasmus University MC, 3015 GD Rotterdam, The Netherlands;
[4]The Abdus Salam International Centre for Theoretical Physics, 34151 Trieste, Italy;
[5]IST Austria, 3400 Klosterneuburg, Austria;
[6]Stem Cell Institute and Lillehei Heart Institute, Department of Medicine, University of Minnesota, Minneapolis, U.S.A.
[7]Department of Pathology, University Medical Center Utrecht, 3584 CX Utrecht, The Netherlands;
[8]Department of Cardiothoracic Surgery, Radboud University Medical Center, Nijmegen, the Netherlands;
[9]International Centre for Genetic Engineering and Biotechnology (ICGEB), Trieste, Italy;
[10]Department of Physiology and Cardiothoracic Surgery, Faculty of Medicine, University of Porto, Porto, Portugal.
[11]School of Cardiovascular Medicine and Sciences, King's College London, London, UK.

A.R. and E.D. contributed equally to this work

Supplementary Figures 1-6
Supplementary Table 1

Supplementary Figures

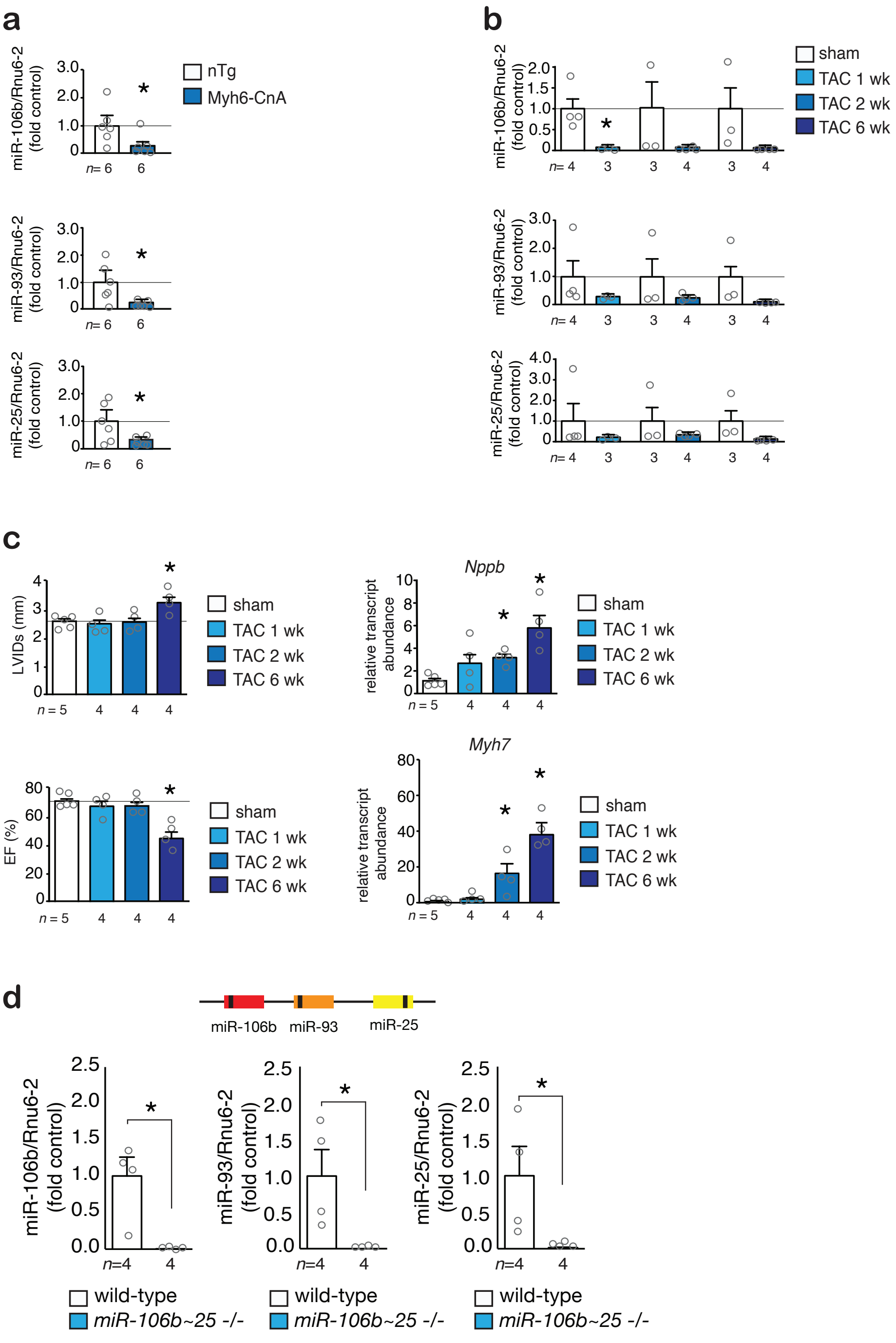

Supplementary Figure 1 | *miR-106b~25* cluster expression. (**a**) Real-time PCR analysis of *miR-106b*, *miR-93* and *miR-25* abundance in hearts from non-transgenic (nTg) or Myh6-CnA transgenic mice or in (**b**) mice subjected to transverse aortic constriction (TAC) for 1, 2 or 6 weeks. (**c**) Indices of cardiac dilation (LVIDs), function (EF) and expression of *Nppb* and *Myh7* in mice subjected to TAC for 1, 2 or 6 weeks. (**d**) Real-time PCR analysis of *miR-106b*, *miR-93* and *miR-25* abundance in hearts of wild-type (WT) or *miR-106b~25* null mice, *n* refers to the number of hearts. *$P < 0.05$ vs corresponding control group (error bars are s.e.m.). Statistical analysis consisted of a two-tailed Student's t-test. Source data are provided as a Source Data file.

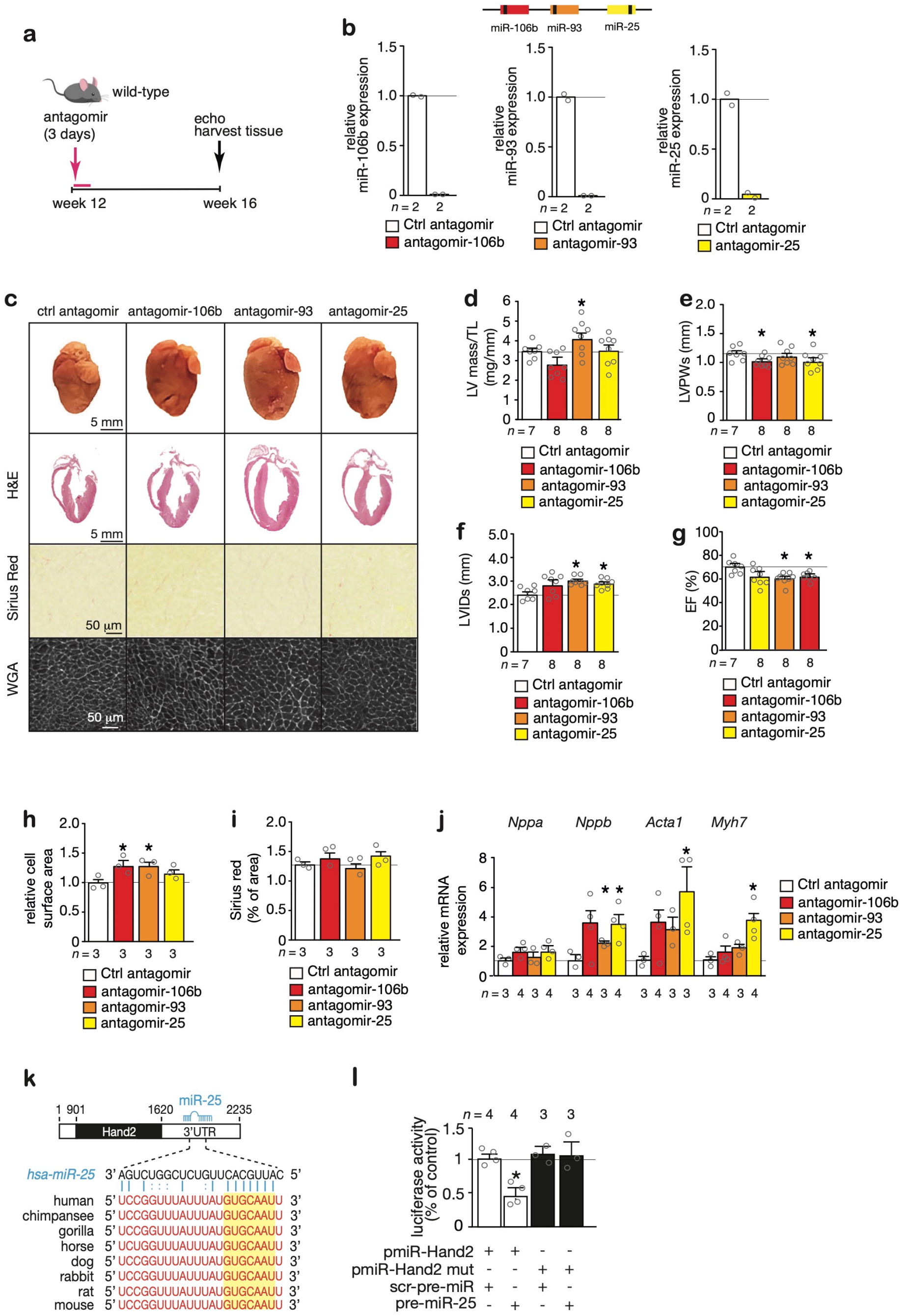

Supplementary Figure 2 | *miR-106b~25* silencing with single antagomirs. (a) Workflow of the study. **(b)** Real-time PCR analysis of *miR-106b*, *miR-93* and *miR-25* expression in hearts from mice receiving control (ctrl) antagomir or antagomir against a specific miRNA. **(c)** Representative images of whole hearts (top panels), H&E-stained sections (second panel), Sirius Red stained sections (third panel) and WGA-stained (fourth panel) histological sections. Quantification of **(d)** LV/BW ratio, **(e)** LVPWs, **(f)** LVIDs, and **(g)** EF of mice that received indicated antagomirs. Quantification of **(h)** cell surface areas by WGA-staining and **(i)** fibrotic area by Sirius Red staining. **(j)** Real-time PCR analysis of *Nppa*, *Nppb*, *Acta1*, and *Myh7*; *n* refers to number of hearts. **(k)** Location and evolutionary conservation of hsa-miR-25 seed region on Hand2. **(l)** Activity assay of luciferase reporter constructs shows the binding of hsa-miR-25 to the 3'UTR of Hand2, *n* refers to number of transfection experiments. *$P < 0.05$ vs corresponding control group (error bars are s.e.m.). Statistical analysis consisted of a two-tailed Student's t-test **(l)** or a One-way ANOVA followed by Dunnett multiple comparison test **(d-g)**. Source data are provided as a Source Data file.

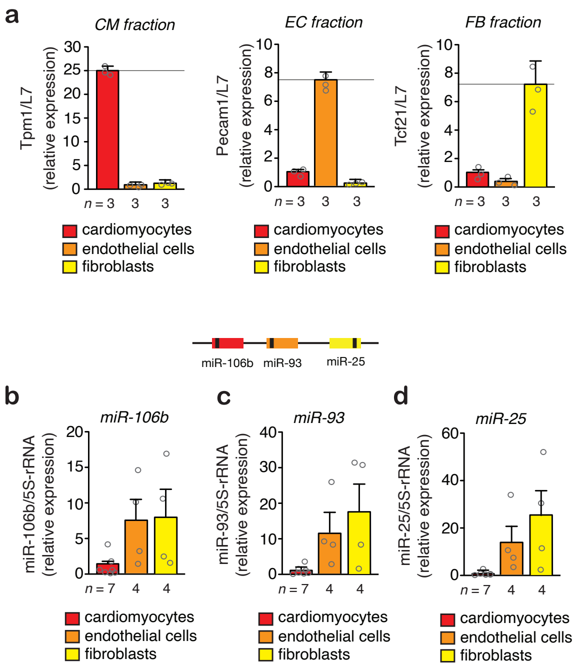

Supplementary Figure 3 I *miR-106b~25* cluster expression in adult mouse heart cells. (a) Real-time PCR analysis of marker genes for Tropomyosin 1 (Tpm1), Platelet endothelial cell adhesion molecule (Pecam1) and Transcription factor 21 (Tcf21) in cardiomyocytes (CMs), endothelial cells (ECs) and fibroblasts (FBs) following enzymatic dissociation of adult mouse hearts followed by column-based magnetic cell isolation. Real-time PCR analysis of *miR-106b*, *miR-93* and *miR-25* abundance in **(b)** CMs, **(c)** ECs or **(d)** FBs, *n* refers to the number of hearts (error bars are s.e.m.). Source data are provided as a Source Data file.

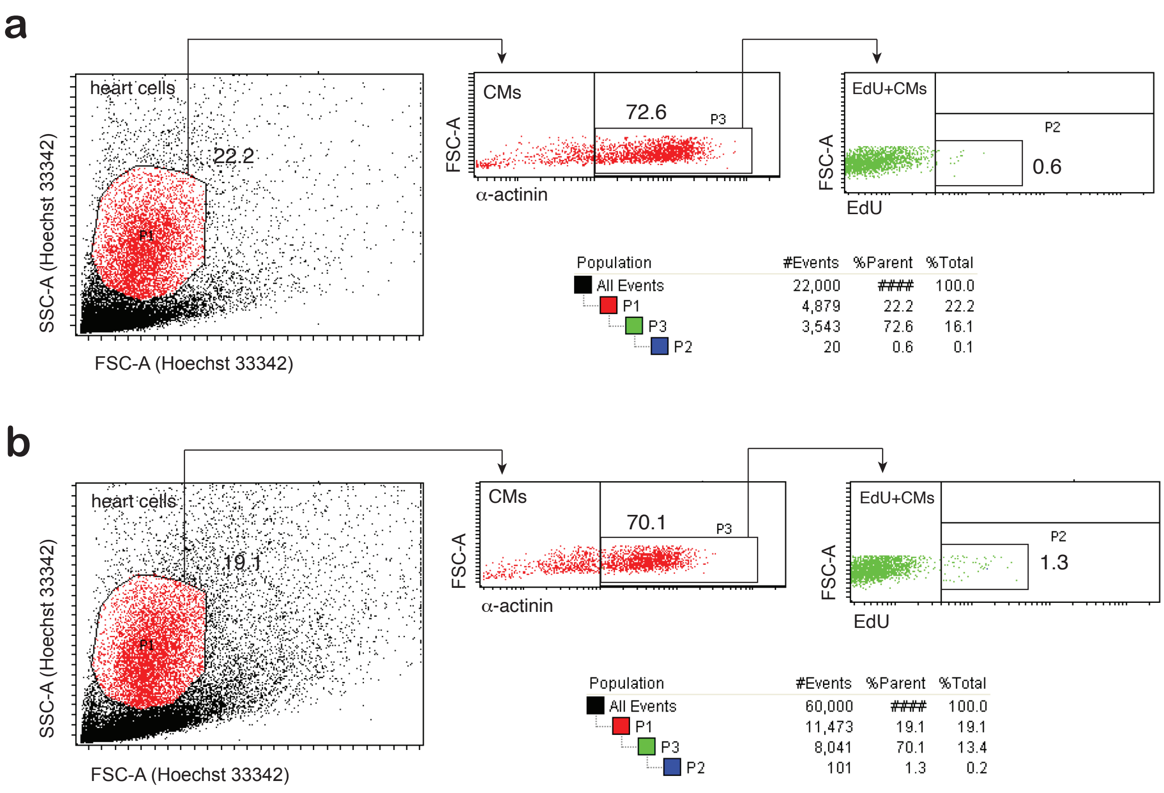

Supplementary Figure 4 I Overexpression of *miR-106b~25* stimulates CM proliferation. Neonatal mice at age p1 received AAV9-MCS or AAV9-miR106b~25, at p10 administered a single EdU injection and 2 days later cardiomyocytes (CMs) from *n=5* hearts in each condition were isolated, pooled and analyzed by flow cytometry. **(a)** Gating strategy for the detection of EdU+CMs within myocardial cells from mice that received AAV9-MCS. **(b)** Gating strategy for the detection of EdU+CMs within myocardial cells from mice that received AAV9-miR106b~25.

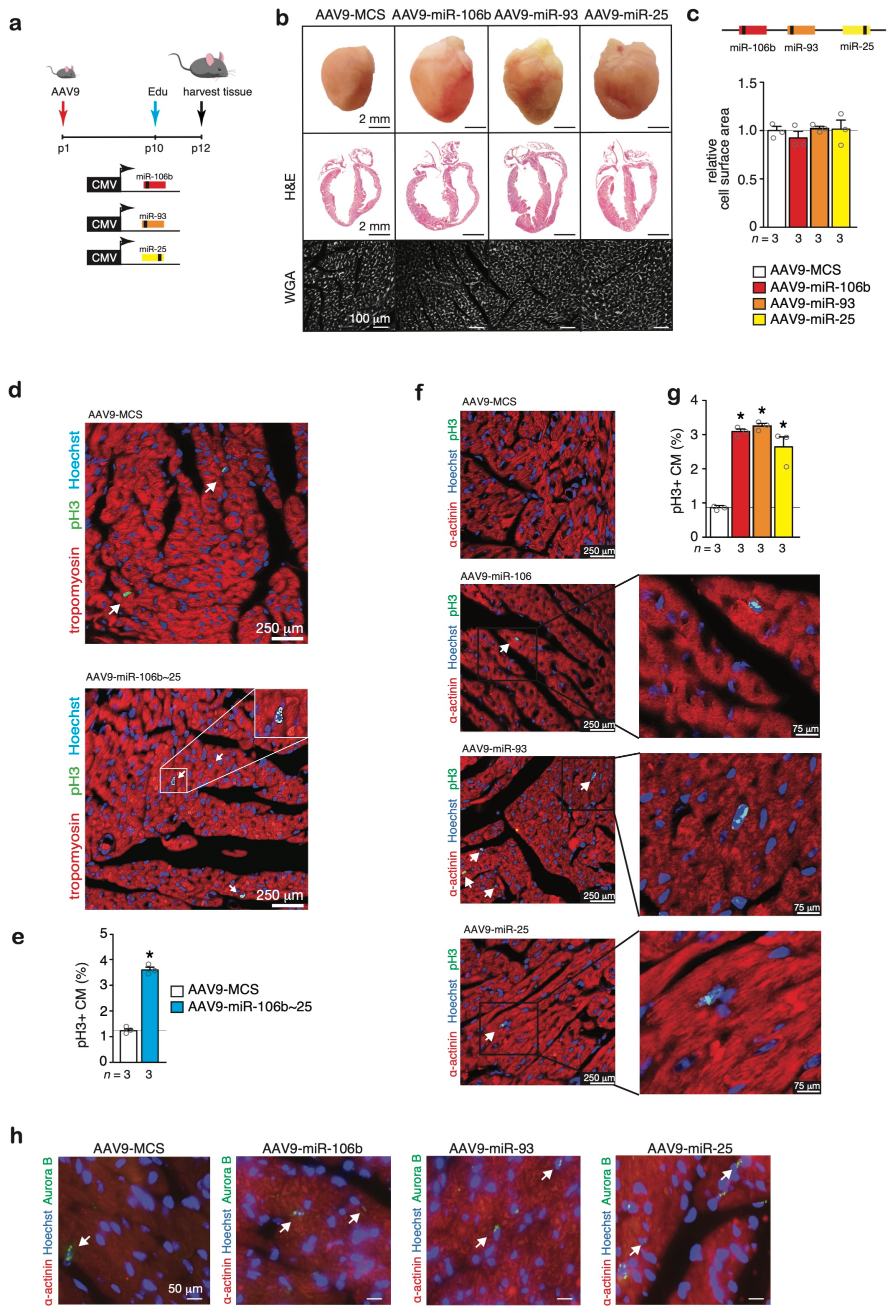

Supplementary Figure 5 | Overexpression of *miR-106b*, *miR-93* or *miR-25* induces cardiac enlargement by stimulating cardiomyocyte proliferation. (a) Design of the study. **(b)** Representative images of whole hearts (top panels), H&E-stained histological sections of four-chamber view (second panel) and WGA-stained (third panel) histological sections. **(c)** Quantification of cell surface areas. **(d)** Representative confocal microscopy images and **(e)** quantification of pH3 positive cardiomyocytes (CMs; α-actinin+, pH3+) in heart sections of mice receiving AAV9-MCS or AAV9-miR-106b~25 and stained for tropomyosin, pH3 and Hoechst. **(f)** Representative confocal microscopy images and **(g)** quantification of the number of pH3 positive CMs (α-actinin+, pH3+) in heart sections of mice receiving AAV9-MCS, AAV9-miR-106b, AAV9-miR-93 or AAV9-miR-25 and stained for α-actinin, pH3 and Hoechst, *n* refers to number of hearts. **(h)** Representative confocal microscopy images of heart sections of mice receiving AAV9-MCS, AAV9-miR-106b, AAV9-miR-93 or AAV9-miR-25 and stained for α-actinin, Aurora B and Hoechst. $^*P < 0.05$ vs corresponding control group (error bars are s.e.m.). Statistical analysis consisted of a two-tailed Student's t-test **(e)** or a One-way ANOVA followed by Dunnett multiple comparison test **(c,g)**. Source data are provided as a Source Data file.

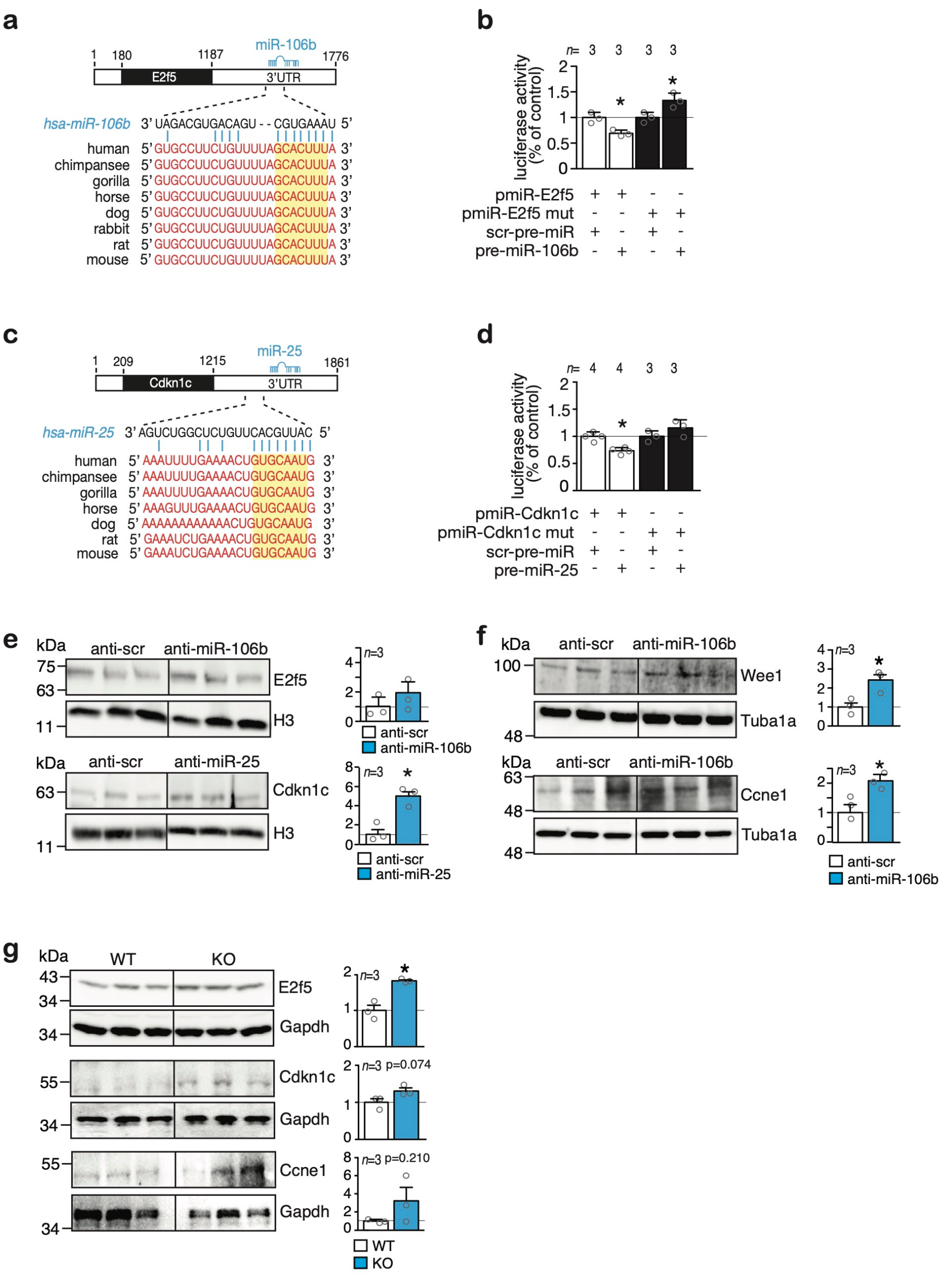

Supplementary Figure 6 | *miR-106b~25* targetome validation. (a) Location and evolutionary conservation of the *hsa-miR-106b* seed region on *E2f5*. **(b)** Activity assay of luciferase reporter constructs shows the binding of *hsa-miR-106b* to the 3'UTR of E2f5. **(c)** Location and evolutionary conservation of the *hsa-miR-25* seed region on *Cdkn1c*. **(d)** Activity assay of luciferase reporter constructs shows the binding of *hsa-miR-25* to the 3'UTR of Cdkn1c, *n* refers to number of transfection experiments. **(e)** Western blot analysis of endogenous E2f5 and Cdkn1c and histone 3 (H3) as a loading control in cardiomyocytes transfected with a control antimiR, or antimiRs for *miR-106b* or *miR-25*. **(f)** Western blot analysis of endogenous Wee1, Ccne1 and Tubulin-a (Tuba1a) as a loading control in cardiomyocytes transfected with a control antimiR, or an antimiR for *miR-106b*. **(g)** Western blot analysis of endogenous E2f5, Cdkn1c, Ccne1 and Gapdh as a loading control in hearts from WT versus *miR-106b~25* KO mice, *n* refers to the number of animals. *$P < 0.05$ vs corresponding control group (error bars are s.e.m.). Statistical analysis consisted of a two-tailed Student's t-test. Source data are provided as a Source Data file.

Supplementary Table 1. real-time PCR primers used in the study

Gene name	Gene identification		sequence
Nppa	NM_008725	FW	TCTTCCTCGTCTTGGCCTTT
		RV	CCAGGTGGTCTAGCAGGTTC
Nppb	NM_008726	FW	TGGGAGGTCACTCCTATCCT
		RV	GGCCATTTCCTCCGACTTT
Acta1	NM_009606	FW	CCGGGAGAAGATGACTCAAA
		RV	GTAGTACGGCC GGAAGCATA
Myh7	NM_080728	FW	CGGACCTTGGAAGACCAGAT
		RV	GACAGC TCCCCATTCTCTGT
Tpm1	NM_001164256	FW	GTATGAAGAGGTGGCCCGTA
		RV	CGAGTTTCAGCCTCCTTCAG
Pecam1	NM_001032378	FW	AACAGAGCTGTTTCCCAAGC
		RV	GTGAAGTTGGCTACAGGTGT
Tcf21	NM_011545	FW	CTTCTCCAGGCTCAAGACCA
		RV	ATAAAGGGCCACGTCAGGTT
Rpl7	NM_011291	FW	GAAGCTCATCTATGAGAAGGC
		RV	AAGACGAAGGAGCTGCAGAAC

All oligos are depicted in 5' --> 3' direction. FW, forward; RV, reverse.
