# Supplementary material for: A microRNA program regulates the balance between cardiomyocyte hyperplasia and hypertrophy and stimulates cardiac regeneration
Source: Nat Commun. 2021 Aug 10;12:4808. doi: 10.1038/s41467-021-25211-4 (PMC8355162; doi:10.1038/s41467-021-25211-4)
Supplement: Supplementary file 3 — Reporting Summary [file 41467_2021_25211_MOESM3_ESM.pdf]

nature portfolio

Corresponding author(s): Leon J. de Windt

Last updated by author(s): Jun 26, 2021

Reporting Summary

Nature Portfolio wishes to improve the reproducibility of the work that we publish. This form provides structure for consistency and transparency in reporting. For further information on Nature Portfolio policies, see our Editorial Policies and the Editorial Policy Checklist.

Statistics

For all statistical analyses, confirm that the following items are present in the figure legend, table legend, main text, or Methods section.

n/a	Confirmed	
☐	☒	The exact sample size (*n*) for each experimental group/condition, given as a discrete number and unit of measurement
☐	☒	A statement on whether measurements were taken from distinct samples or whether the same sample was measured repeatedly
☐	☒	The statistical test(s) used AND whether they are one- or two-sided<br>*Only common tests should be described solely by name; describe more complex techniques in the Methods section.*
☐	☒	A description of all covariates tested
☐	☒	A description of any assumptions or corrections, such as tests of normality and adjustment for multiple comparisons
☐	☒	A full description of the statistical parameters including central tendency (e.g. means) or other basic estimates (e.g. regression coefficient) AND variation (e.g. standard deviation) or associated estimates of uncertainty (e.g. confidence intervals)
☐	☒	For null hypothesis testing, the test statistic (e.g. *F*, *t*, *r*) with confidence intervals, effect sizes, degrees of freedom and *P* value noted<br>*Give P values as exact values whenever suitable.*
☒	☐	For Bayesian analysis, information on the choice of priors and Markov chain Monte Carlo settings
☒	☐	For hierarchical and complex designs, identification of the appropriate level for tests and full reporting of outcomes
☐	☒	Estimates of effect sizes (e.g. Cohen's *d*, Pearson's *r*), indicating how they were calculated

Our web collection on statistics for biologists contains articles on many of the points above.

Software and code

Policy information about availability of computer code

Data collection	no software was used for data collection
Data analysis	Open software used was:<br><br>FACSDiva software v6.1.3<br>HiSeq software v2.2.68 (HiSeq2000)<br>FASTQC software v0.11.9<br>Cutadapt software v3.4<br>SciPy v0.18.1<br>TargetScanMouse release 7.1<br>STRING database v10.5<br>NetworkX v1.11 (Python)<br>Cytoscape v3.4.0 (Python)<br>Prism software v9.1.2 (GraphPad)<br>STAR v2.4.0d<br>Bioconductor package EdgeR release 3.13

For manuscripts utilizing custom algorithms or software that are central to the research but not yet described in published literature, software must be made available to editors and reviewers. We strongly encourage code deposition in a community repository (e.g. GitHub). See the Nature Portfolio guidelines for submitting code & software for further information.

Data

Policy information about availability of data

All manuscripts must include a data availability statement. This statement should provide the following information, where applicable:

- Accession codes, unique identifiers, or web links for publicly available datasets
- A description of any restrictions on data availability
- For clinical datasets or third party data, please ensure that the statement adheres to our policy

The following Data availability section was included in the Methods section: Data availability. Published resources evaluated included HomoloGene NCBI database (https://www.ncbi.nlm.nih.gov/homologene) and STRING (https://string-db.org/). The source data that support the findings in this study are available within the article and its Supplementary Information files, and from the corresponding author upon request. Raw and processed RNAseq data generated in this study have been deposited at the GEO database under accession code: GSE178867.

Field-specific reporting

Please select the one below that is the best fit for your research. If you are not sure, read the appropriate sections before making your selection.

- [x] Life sciences
- [ ] Behavioural & social sciences
- [ ] Ecological, evolutionary & environmental sciences

For a reference copy of the document with all sections, see nature.com/documents/nr-reporting-summary-flat.pdf

Life sciences study design

All studies must disclose on these points even when the disclosure is negative.

Sample size	For animal experiments, sample size was determined by a power calculation based upon an echocardiographic effect size. For other experiments no sample side was determined.
Data exclusions	No data was excluded from the analysis in this study.
Replication	A statement on Reproducibility was included in the Methods section: Statistics and Reproducibility. Results shown for images or blots were repeated independently at least once with similar results.
Randomization	For animal experiments, Randomization of subjects to experimental groups was based on a single sequence of random assignments. For other experiments, no randomization took place.
Blinding	For animal experiments, animal caretakers blinded investigators to group allocation during the experiment and/or when assessing the outcome. For other experiments no blinding occurred.

Reporting for specific materials, systems and methods

We require information from authors about some types of materials, experimental systems and methods used in many studies. Here, indicate whether each material, system or method listed is relevant to your study. If you are not sure if a list item applies to your research, read the appropriate section before selecting a response.

Materials & experimental systems

n/a	Involved in the study	
[ ]	[x]	Antibodies
[x]	[ ]	Eukaryotic cell lines
[x]	[ ]	Palaeontology and archaeology
[ ]	[x]	Animals and other organisms
[ ]	[x]	Human research participants
[x]	[ ]	Clinical data
[x]	[ ]	Dual use research of concern

Methods

n/a	Involved in the study	
[x]	[ ]	ChIP-seq
[ ]	[x]	Flow cytometry
[x]	[ ]	MRI-based neuroimaging

Antibodies

Antibodies used

primary antibodies

1. mouse monoclonal antibody [EA-53] to sarcomeric alpha-actinin (1:100, Abcam ab9465)
2. FITC-labelled rabbit polyclonal antibody against wheat-germ-agglutinin (WGA) (1:100, Sigma Aldrich T4144)
3. rabbit polyclonal anti-phospho-Histone H3 (Ser10) (1:100, Sigma-Millipore 06-570)
4. rabbit polyclonal anti-Aurora B (1:100, Abcam ab2254)

5. rabbit polyclonal anti-PCM-1 antibody (0.4 mg/µl in blocking solution, Sigma-Aldrich HPA023370)
6. rabbit polyclonal antibody anti-p57 (H-91) (1:500, SantaCruz sc-8298)
7. rabbit polyclonal antibody anti-E2f5 (1:500, Abcam ab22855)
8. rabbit polyclonal antibody anti-E2F5 (E-19) (1:500, SantaCruz sc-999)
9. rabbit monoclonal antibody anti-Cyclin E1 (D7T3U) (1:500, Cell Signaling Technology #20808)
10. rabbit polyclonal antibody anti-Wee1 (1:500, Cell Signaling Technology #4936)
11. rabbit polyclonal antibody anti-Mef2d (1:500, Abcam ab104515)
12. mouse monoclonal anti-GAPDH (1:5000, Millipore, MAB374 clone 6C5)
13. mouse monoclonal anti-alpha-Tubulin (1:5000, Sigma-Aldrich T6074)
14. rabbit polyclonal anti-Histone H3 (1:5000, Cell Signaling Technology 9715S)

secondary antibodies

1. goat anti-mouse IgG secondary antibody Alexa Fluor-488 conjugated (1:100, ThermoFisher A-11001)
2. donkey anti-rabbit IgG secondary antibody Alexa Fluor-555 conjugated (1:100, ThermoFisher A32794)
3. goat anti-mouse IgG secondary antibody Alexa Fluor-647 conjugated (1:100, Thermofisher A32728)
4. goat anti-rabbit IgG secondary antibody Alexa Fluor-488 conjugated (4 mg/µl in blocking solution, ThermoFisher A32731)
5. polyclonal swine anti-rabbit immunoglobulins/HRP (1:10.000, DAKO P0399)
6. polyclonal rabbit anti-mouse immunoglobulins/HRP (1:10.000, DAKO P0161)

Validation

Validation from supplier website: primary antibodies were validated to react to mouse proteins by immunohistochemistry and immunoblotting. FITC-labelled antibody against wheat-germ-agglutinin (Sigma), polyclonal anti-phospho-Histone H3 (Ser10) (Sigma-Millipore), rabbit polyclonal anti-Aurora B (Abcam), rabbit polyclonal antibody anti-E2f5 (Abcam), rabbit monoclonal antibody anti-Cyclin E1 (Cell Signaling Technology), mouse monoclonal anti-GAPDH (Millipore), rabbit polyclonal anti-Histone H3 (Cell Signaling Technology).

Validation from supplier website: primary antibodies were validated to react with human or rat proteins by immunohistochemistry and immunoblotting. Mouse monoclonal anti-sarcomeric alpha-actinin, rabbit polyclonal anti-PCM-1 antibody, rabbit polyclonal antibody anti-Wee1, rabbit polyclonal antibody anti-Mef2d, mouse monoclonal anti-alpha-Tubulin.

Variation from supplier website: All primary antibodies were validated to react with murine family counterparts though in silico analysis of relevant immunogens, with a minimum of 95% homology.

Validation from supplier website: product discontinued. polyclonal antibody anti-p57 (H-91) (SantaCruz sc-8298), rabbit polyclonal antibody anti-E2F5 (E-19) (SantaCruz sc-999).

Variation from supplier website: All primary antibodies were validated to react with murine family counterparts though in silico analysis of relevant immunogens, with a minimum of 95% homology.

Animals and other organisms

Policy information about studies involving animals; ARRIVE guidelines recommended for reporting animal research

Laboratory animals

1. Mice homozygous null for the mirc3 cluster (Mirc3tm1.1Tyj/J, Stock No: 008460) were maintained in a B6SV129F1 background. Both male and female mice of 3-6 months of age were used in this study.
2. 3-6 month old male calcineurin transgenic mice in a B6SV129F1 background were used
3. male and female CD1 wild-type mice ranging between postnatal (p) day 0 and p56 were used
4. male and female B6SV129F1 wild-type mice of 3-6 months of age were used
5. Cre-responsive Rosa26-TdTomato (R26-TdT) reporter mice were crossbred with Myh6-mER-Cre-mER mice to generate Myh6-mER-Cre-mER R26-TdT mice in a B6SV129F1 background and both male and female mice of 3-6 months of age were used.

Wild animals

no wild animals were used in the study

Field-collected samples

no field-collected samples were used in the study.

Ethics oversight

All animal studies were performed in accordance with local institutional guidelines and regulations from Medanex Inc., the International Centre for Genetic Engineering and Biotechnology (ICGEB) and the University of Minnesota.

Note that full information on the approval of the study protocol must also be provided in the manuscript.

Human research participants

Policy information about studies involving human research participants

Population characteristics

Samples included tissue from the left ventricular free wall of patients with end-stage heart failure secondary to ischemic

	heart disease. Control tissue was taken from the left ventricular free wall of refused donor hearts.
Recruitment	Samples were obtained by the Biobank at University Medical Center Utrecht, Utrecht the Netherlands or the Biobank at the University Hospital Hamburg, Hamburg Germany
Ethics oversight	Approval for studies on human tissue samples was obtained from the Medical Ethics Committee of the University Medical Center Utrecht, The Netherlands, and by the Ethical Committee of the University Hospital Hamburg, Germany

Note that full information on the approval of the study protocol must also be provided in the manuscript.

Flow Cytometry

Plots

Confirm that:

- [x] The axis labels state the marker and fluorochrome used (e.g. CD4-FITC).
- [x] The axis scales are clearly visible. Include numbers along axes only for bottom left plot of group (a 'group' is an analysis of identical markers).
- [x] All plots are contour plots with outliers or pseudocolor plots.
- [x] A numerical value for number of cells or percentage (with statistics) is provided.

Methodology

Sample preparation	Sample preparation was described in the Methods section.
Instrument	BD FACSCelesta cell analyser
Software	FACSDiva version 6.1.3
Cell population abundance	22.000 cells for experiment in suppl. fig. 4a; 60.000 cells for experiment in suppl. fig. 4b
Gating strategy	Cells were first gated based on light scatter properties based on cells size (FSC-A) and granularity (SSC-A) and width parameter on forward scatter was used to gate out doublets. Cardiomyocytes were gated based upon positive staining for alpha-actinin and FSC-A. EdU+ cardiomyocytes were gated based upon positive EdU staining and FSC-A. The gating strategy is provided in-figure in Suppl. Fig. 4

- [x] Tick this box to confirm that a figure exemplifying the gating strategy is provided in the Supplementary Information.
